# Supplementary material for: Disproportionate impacts of COVID-19 on marginalized and minoritized early-career academic scientists
Source: PLoS One. 2022 Sep 13;17(9):e0274278. doi: 10.1371/journal.pone.0274278 (PMC9469975; doi:10.1371/journal.pone.0274278)
Supplement: S1 File — (DOCX) [file pone.0274278.s001.docx]

**Supporting Information**

**Participant Database**

As the basis of our study population, we selected four STEM fields where racial minority and/or gender representation are low: Biology, Economics, Physics, and Psychology. Using the 2011 National Research Council S-rankings [1], which provides an assessment of doctoral program quality based on faculty ratings, we divided all US university departments in the four fields into terciles reflecting top, middle, and bottom tier rankings. After excluding the investigator institutions (University of Michigan and Michigan State University), we randomly selected 10 institutions in each tercile from each academic field, resulting in 120 departments. We added 1 minority serving institution (MSI) to any tercile that did not have at least one already randomly selected to increase the number of racial minority participants in our pool. Specifically, one in tercile 3 in Economics, one in tercile 1 in Biology, one in tercile 2 in Biology, and one in tercile 1 in Psychology (124 departments total across the four disciplines). We developed our participant pool by requesting email addresses of doctoral students, postdoctoral fellows, and assistant professors from department leadership (chairs and chief administrators) or compiling this information from department websites. If emails could not be obtained from department leadership or websites, we replaced the department with a randomly selected alternative in the same tier and academic field. We excluded terminal master’s students, non-tenure track faculty, assistant professors, and full professors. Our final participant pool in the database consisted of 10,658 persons (8,750 doctoral students, 1,114 postdocs, and 789 assistant professors). Within our sample, *n* = 66 participants were no longer in the department or university we recruited them from. Of those, we retained *n* = 53 participants because they were in our target fields while *n* = 13 participants were no longer in one of the four target fields and were excluded from analysis. Finally, *n* = 679 participants did not report their current university; these observations were retained if they were still in a target field. Our final sample consisted of *N* = 3,243 participants included in the analyses presented in this paper.

| Table S1. The numbers of participants invited to participate (population), completing the survey (sample), and the response rate by academic field and career stage. | | | | | |
| --- | --- | --- | --- | --- | --- |
| Field |  | Doctoral Students | Postdocs | Assistant Professors | Total |
| Biology | Sample | 646 | 145 | 64 | 855 |
|  | Population | 2,110 | 436 | 219 | 2,765 |
|  | Response rate | 31% | 33% | 29% | 31% |
| Economics | Sample | 573 | 10 | 41 | 624 |
|  | Population | 2,010 | 17 | 222 | 2,249 |
|  | Response rate | 29% | 59% | 18% | 28% |
| Physics | Sample | 714 | 103 | 51 | 868 |
|  | Population | 2,807 | 468 | 179 | 3,454 |
|  | Response rate | 25% | 22% | 28% | 25% |
| Psychology | Sample | 754 | 77 | 65 | 896 |
|  | Population | 1,823 | 193 | 169 | 2,185 |
|  | Response rate | 41% | 40% | 38% | 41% |
| Total | Sample | 2,687 | 335 | 221 | 3,243 |
|  | Population | 8,750 | 1,114 | 789 | 10,653 |
|  | Response rate | 31% | 30% | 28% | 30% |

**Participants and Exclusion Criteria**

Participants were recruited via email through the Qualtrics survey distribution platform and received a mailed check as participation incentive of $35.00, $25.00, or $20.00 depending on when they completed the survey. Participants who completed the survey within seven days of the survey launch received a $35.00 check; if they completed the survey between 8 and 28 days of the launch, they received $25.00; and those who completed the survey between 21 and 28 days of the launch received $20.00. The survey was open between April and May 2021. Only participants who stated that they were either doctoral students, postdoctoral scholars, or assistant professors were able to complete the survey; those who responded with any other career stage were redirected out of the survey. To be included in the analyses presented in this paper, participants had to complete the relevant COVID-19 and demographic questions, which represented at least 94% survey completion, report being at a US institution, and in one of our four target fields (*n* = 3,243 out of *N* = 3,579 total responses; mean age = 29.6 years old, *SD* = 5.1). Table S1 details the total number of respondents by both academic field and career stage as well as the number of respondents included in the analyses. With the exception of career stage, none of the survey questions were mandatory. As all the predictors were socio-demographic characteristics, rather than imputing missing data, we used listwise deletion.

**Socio-demographic variables**

Table S2 reports the number of participants in each category within socio-demographic status. For statuses where we combined groups for data analyses, we report both the aggregated groupings used for the data analyses (“analytic group”), and the disaggregated groups. Because only career stage required a response on the survey, the number of participants for each socio-demographic status varies.

Participants self-reported their gender identity as woman; man; gender non-binary, genderqueer, and/or gender fluid; or “I use a different label.” We combined participants who self-identified as woman, gender non-binary, genderqueer, or gender fluid into a single analytic group (“women and non-binary”) because of the small number of individuals identifying as gender non-binary, genderqueer, and/or gender fluid and because their responses to work and life impacts tended to be in the same direction as women’s responses (Table S2). While non-binary participants did significantly differ compared to both women and men on the impact that mental health symptoms had on work, the difference was still in the same direction for non-binary participants (B = 0.36, *p* = .027) and women (B = 0.23, *p* < .001) when compared to men.

The parental caregiving measure was created using information from two variables. Participants who reported that they did not have children in their household were categorized as “non-parents.” Participants with one or more children were asked “who has the majority responsibility for taking care of the child(ren) in your household?” Those who indicated that they had the majority responsibility were categorized as “primary caregivers” and those who reported that they share responsibility with one or more adults, or one or more other adults has the primary responsibility, were categorized as “non-primary caregivers.”

To assess racial/ethnic identity, participants were asked to “Select all of the following racial/ethnic categories with which you identify” and response options were Asian, Asian American, or Pacific Islander; Black or African American; Hispanic or Latina(o) or Latinx; Middle Eastern or North African; Native American, American Indian, Alaska Native, First Nations or other Indigenous category; White or Caucasian; or “None of these categories describes me. I identify as” with an option to write-in a response. We combined Black/African American, Hispanic/Latina(o)/Latinx, Middle Eastern/North African, and Native American/American Indian/Alaska Native/First Nations/other Indigenous into a single category of underrepresented minority (URM) as these groups have similar experiences of underrepresentation and negative intellectual stereotypes within the academy [2,3]. In analyses, we used two dummy variables, URM (vs. White) and Asian/Asian American (vs. White) to assess racial group differences. We distinguished between Asian/Asian American and URM respondents as Asians/Asian Americans are not statistically underrepresented in academic science [2]; however, we distinguish them from White academics given that they still experience marginalization within the academy [3]. For those who identified as White and Asian, we included them in the Asian category.

To assess disability status, participants were asked, “Do any of the following make it difficult for you to do your work?” followed by a list of five types of impairments. Participants were able to select multiple responses. Participants were coded as having a disability if they self-identified as having at least one of the following: physical difficulties (e.g., physical movement, speaking, hearing difficulties, vision difficulties); mental health issues (e.g., depression, bipolar disorder); chronic illness (e.g., Crohn's disease, cancer); learning, reading, or writing difficulties (e.g., dyslexia); or neurological differences (e.g., Autism Spectrum, ADHD, dyspraxia). All other participants were coded as not having a disability.

For sexual identity, participants were asked “Which of the following describes your sexual identity?” with the response options: straight/heterosexual, lesbian, gay, bisexual, pansexual, queer, asexual, or “I use a different label” with an option to write-in their response. Participants who responded as lesbian, gay, bisexual, pansexual, queer, asexual, or filled in with a different label were coded as sexual minority and compared to those who responded as heterosexual.

To assess first generation college status, participants were asked “Are you a first-generation college student?” with the response options yes, no, or “I don’t know”. Participants who responded “Yes” were compared to those who responded “No.” Those who responded “I don’t know” were treated as missing data and not included in the analysis.

For career stage, participants were asked “What is your current academic position?” and selected one from the following list: Master's Student (terminal); PhD Student; Postdoc; Teaching Professor, Adjunct Professor, or Lecturer; Tenure-track Assistant Professor; Tenure-track or Tenured Associate Professor; Other position. This question was the only survey item that required a forced response as it determined eligibility for participation in the study. Participants who did not select either “PhD Student,” “Postdoc,” or “Tenure-track Assistant Professor” were redirected out of the survey as those were the three academic positions of interest for this study.

Finally, for academic field, participants were asked “What is your academic field?” with the response options: biology, economics, physics, psychology, and other with an option to write-in their response. The majority of the “other” responses could be recoded into the four target fields (*n* = 53) while *n* = 13 responses were excluded as they described being in a field outside of the four target fields. Finally, *n* = 1 participant did not respond to this item; their field was imputed with the information we obtained from the participant database.

| Table S2. Number of participants who self-identified in each of the socio-demographic statuses included in the analysis. | | |
| --- | --- | --- |
| Socio-  demographic status | Analytic Groups  *Disaggregated groups* | *N* (%) |
| Gender | Man | 1,564 (48.2%) |
|  | Woman and non-binary | 1,642 (50.6%) |
|  | *Woman* | *1,577 (48.6%)* |
|  | *Genderqueer, gender non-binary, and/or gender fluid* | *65 (2.0%)* |
| Parental caregiving | Primary caregiver | 82 (2.5%) |
|  | Non-primary caregiver | 272 (8.4%) |
|  | *Shared responsibility equally with child’s parent* | *185 (5.7%)* |
|  | *Shared responsibility with other adult (not the child’s parent)* | *14 (0.4%)* |
|  | *Spouse or partner is primary caregiver* | *67 (2.1%)* |
|  | *Another adult who is not their spouse/partner or the child’s other parent is the primary caregiver* | *6 (0.2%)* |
|  | Non-parent | 2,878 (88.7%) |
| Race† | White | 1,688 (52.1%) |
|  | Asian/Asian American | 984 (30.3%) |
|  | URM | 534 (16.5%) |
|  | *Black or African American* | *125 (3.9%)* |
|  | *Hispanic or Latina/o/x* | *306 (9.4%)* |
|  | *Middle Eastern or North African* | *110 (3.4%)* |
|  | *Native American, American Indian, or other Indigenous group* | *25 (0.8%)* |
| Sexual identity | Heterosexual | 2,586 (79.7%) |
|  | Sexual minority | 572 (17.6%) |
|  | *Lesbian* | *42 (1.3 %)* |
|  | *Gay* | *103 (3.2%)* |
|  | *Bisexual* | *262 (8.1%)* |
|  | *Pansexual* | *50 (1.5%)* |
|  | *Queer* | *71 (2.2%)* |
|  | *Asexual* | *25 (0.8%)* |
|  | *Demisexual* | *6 (0.2%)* |
|  | *Other label* | *13 (0.4%)* |
| First generation college student | Not a first gen college student | 2,324 (71.7%) |
|  | First gen college student | 859 (26.5%) |
| Disability Status† | No disability | 1,662 (51.2%) |
|  | Disability | 1,487 (45.9%) |
|  | *Physical difficulties* | *202 (6.2%)* |
|  | *Mental illness* | *1,203 (19.3%)* |
|  | *Chronic illness* | *170 (5.2%)* |
|  | *Learning, reading, and/or writing difficulties* | *108 (3.3%)* |
|  | *Neurological differences* | *287 (8.8%)* |
|  | *Other disability* | *103 (3.2%)* |
| Career Stage | Doctoral student | 2,687 (82.5%) |
|  | Postdoctoral scholar | 335 (10.3%) |
|  | Assistant professor | 221 (6.8%) |
| *Note.* For socio-demographic statuses that were aggregated for analysis, we present the *N*’s for both the aggregated analytic groupings and the disaggregated groups.  †Participants were instructed to “select all that apply for these demographic items. Therefore, the total for race and disability is greater than 100%. | | |

| Table S3. Unstandardized regression coefficients, standard errors, *p*-values, and *n*’s for change in research progress by socio-demographic status for the full sample, doctoral student subsample, and postdoctoral scholar and assistant professor subsample. | | | | | | | | | |
| --- | --- | --- | --- | --- | --- | --- | --- | --- | --- |
|  | Full sample  *R^2^* = .04, *df* = 15, 2,960 | | | Doctoral Students  *R^2^* = .03, *df* = 13, 2,462 | | | Postdoctoral Scholars and Assistant Professors  *R^2^* = .09, *df* = 14, 485 | | |
|  | B (SE) | *p* | *n* | B (SE) | *p* | *n* | B (SE) | *p* | *n* |
| Woman or non-binary^1^ | -0.03 (.05) | .485 | 1,529 (1,447) | -0.05 (.05) | .376 | 1,315 (1,161) | 0.04 (.10) | .719 | 214 (286) |
| Primary caregiver^2^ | -0.72 (.13) | <.001 | 76 (2,640) | -0.68 (.18) | <.001 | 40 (2,321) | -0.75 (.19) | <.001 | 36 (319) |
| Non-primary caregiver^2^ | -0.22 (.08) | .007 | 260 | -0.15 (.11) | .172 | 115 | -0.29 (.11) | .010 | 145 |
| Asian^3^ | 0.02 (.05) | .730 | 888 (1,581) | 0.01 (.05) | .813 | 775 (1,266) | 0.05 (.12) | .665 | 113 (315) |
| URM^3^ | -0.002 (.06) | .966 | 507 | 0.004 (.06) | .946 | 435 | -0.08 (.14) | .565 | 72 |
| Sexual minority^4^ | -0.04 (.06) | .416 | 547 (2,429) | -0.04 (.06) | .544 | 492 (1,984) | -0.11 (.16) | .497 | 55 (445) |
| First generation college^5^ | 0.02(.05) | .747 | 819 (2,157) | 0.002 (.05) | .976 | 664 (1,812) | 0.08 (.10) | .403 | 155 (345) |
| Disability^6^ | -0.24 (.04) | <.001 | 1,412 (1,564) | -0.24 (.05) | <.001 | 1,236 (1,240) | -0.23 (.10) | .018 | 176 (324) |
| Postdoc^7^ | -0.03 (.07) | .676 | 299 (2,476) | – | – | – | – | – | – |
| Assistant Professor^7^ | -0.21 (.09) | .019 | 201 | – | – | – | -0.20 (.11) | .071 | 201 (299) |
| Tier 2^8^ | 0.10 (.05) | .034 | 922 (1,432) | 0.12 (.05) | .024 | 763 (1,198) | -0.01 (.11) | .898 | 159 (234) |
| Tier 3^8^ | 0.14 (.05) | .011 | 622 | 0.12 (.06) | .043 | 515 | 0.24 (.13) | .064 | 107 |
| Biology^9^ | -0.23 (.06) | <.001 | 785 (840) | -0.25 (.06) | <.001 | 596 (711) | -0.12 (.12) | .323 | 189 (129) |
| Economics^9^ | 0.07 (.06) | .295 | 578 | 0.02 (.07) | .807 | 531 | 0.50 (.18) | .007 | 47 |
| Physics^9^ | -0.10 (.06) | .092 | 773 | -0.14 (.07) | .039 | 638 | 0.09 (.13) | .483 | 135 |
| *Note.* Referent groups *n*s given parenthetically under focal group n; reference groups are as follows: ^1^ Men, ^2^ Non-parent, ^3^ White, ^4^ heterosexual, ^5^ not first generation, ^6^ no disability, ^7^ doctoral student, ^8^ tier 1, and ^9^ psychology. | | | | | | | | | |

| Table S4. Unstandardized regression coefficients, standard errors, *p*-values, and *n*’s for change in workload by socio-demographic status for the full sample, doctoral student subsample, and postdoctoral scholar and assistant professor subsample. | | | | | | | | | |
| --- | --- | --- | --- | --- | --- | --- | --- | --- | --- |
|  | Full sample  *R^2^* = .05, *df* = 15, 2,964 | | | Doctoral Students  *R^2^* = .04, *df* = 13, 2,470 | | | Postdoctoral Scholars and Assistant Professors  *R^2^* = .13, *df* = 14, 484 | | |
|  | B (SE) | *p* | *n* | B (SE) | *p* | *n* | B (SE) | *p* | *n* |
| Woman or non-binary^1^ | 0.11 (.04) | .002 | 1,529 (1,451) | 0.14 (.04) | .001 | 1,316 (1,165) | -0.03 (.08) | .692 | 213 (286) |
| Primary caregiver^2^ | -0.13 (.11) | .220 | 75 (2,645) | -0.26 (.14) | .068 | 40 (2,326) | 0.10 (.15) | .519 | 35 (319) |
| Non-primary caregiver^2^ | -0.04 (.06) | .544 | 260 | -0.13 (.09) | .124 | 115 | 0.09 (.09) | .305 | 145 |
| Asian^3^ | -0.09 (.04) | .021 | 889 (1,585) | -0.08 (.04) | .070 | 776 (1,271) | -0.14 (.09) | .125 | 113 (314) |
| URM^3^ | 0.07 (.05) | .109 | 506 | 0.10 (.05) | .055 | 434 | 0.02 (.11) | .877 | 72 |
| Sexual minority^4^ | -0.01 (.04) | .793 | 547 (2,433) | -0.01 (.05) | .880 | 492 (1,989) | -0.01 (.12) | .907 | 55 (444) |
| First generation college^5^ | 0.06 (.04) | .105 | 817 (2,163) | 0.04 (.04) | .390 | 663 (1,818) | 0.16 (.08) | .049 | 154 (345) |
| Disability^6^ | 0.10 (.03) | .003 | 1,412 (1,568) | 0.10 (.04) | .007 | 1,237 (1,244) | 0.11 (.08) | .178 | 175 (324) |
| Postdoc^7^ | -0.07 (.06) | .196 | 298 (2,481) | – | – | – | – | – | – |
| Assistant Professor^7^ | 0.42 (.07) | <.001 | 201 | – | – | – | 0.53 (.09) | <.001 | 201 (298) |
| Tier 2^8^ | 0.11 (.04) | .004 | 924 (1,434) | 0.12 (.04) | .006 | 765 (1,201) | 0.07 (.09) | .398 | 159 (233) |
| Tier 3^8^ | 0.16 (.04) | <.001 | 622 | 0.20 (.05) | <.001 | 515 | -0.02 (.10) | .812 | 107 |
| Biology^9^ | -0.12 (.04) | .006 | 782 (841) | -0.12 (.05) | .017 | 594 (712) | -0.13 (.10) | .172 | 188 (129) |
| Economics^9^ | 0.03 (.05) | .614 | 581 | 0.06 (.05) | .287 | 534 | -0.21 (.15) | .161 | 47 |
| Physics^9^ | -0.17 (.05) | .001 | 776 | -0.16 (.05) | .004 | 641 | -0.17 (.11) | .102 | 135 |
| *Note.* Referent groups *n*s given parenthetically under focal group n; reference groups are as follows: ^1^ Men, ^2^ Non-parent, ^3^ White, ^4^ heterosexual, ^5^ not first generation, ^6^ no disability, ^7^ doctoral student, ^8^ tier 1, and ^9^ psychology. | | | | | | | | | |

| Table S5. Unstandardized regression coefficients, standard errors, *p*-values, and *n*’s for change in concern about career advancement by socio-demographic status for the full sample, doctoral student subsample, and postdoctoral scholar and assistant professor subsample. | | | | | | | | | |
| --- | --- | --- | --- | --- | --- | --- | --- | --- | --- |
|  | Full sample  *R^2^* = .03, *df* = 15, 2,965 | | | Doctoral Students  *R^2^* = .02, *df* = 13, 2,467 | | | Postdoctoral Scholars and Assistant Professors  *R^2^* = .11, *df* = 14, 485 | | |
|  | B (SE) | *p* | *n* | B (SE) | *p* | *n* | B (SE) | *p* | *n* |
| Woman or non-binary^1^ | 0.03 (.04) | .460 | 1,532 (1,449) | 0.001 (.04) | .997 | 1,318 (1,163) | 0.15 (.09) | .104 | 214 (286) |
| Primary caregiver^2^ | 0.12 (.11) | .264 | 76 (2,645) | 0.02 (.15) | .866 | 40 (2,326) | 0.17 (.17) | .316 | 36 (319) |
| Non-primary caregiver^2^ | 0.03 (.07) | .623 | 260 | 0.03 (.09) | .702 | 115 | 0.05 (.10) | .623 | 145 |
| Asian^3^ | 0.06 (.04) | .129 | 892 (1,583) | 0.09 (.04) | .029 | 779 (1,268) | -0.15 (.11) | .148 | 113 (315) |
| URM^3^ | 0.12 (.05) | .013 | 506 | 0.10 (.05) | .060 | 434 | 0.26 (.13) | .036 | 72 |
| Sexual minority^4^ | 0.04 (.05) | .394 | 547 (2,434) | 0.05 (.05) | .303 | 492 (1,989) | 0.01 (.14) | .928 | 55 (445) |
| First generation college^5^ | -0.02 (.04) | .575 | 819 (2,162) | -0.02 (.04) | .620 | 664 (1,817) | -0.05 (.09) | .601 | 155 (345) |
| Disability^6^ | 0.21 (.04) | <.001 | 1,411 (1,570) | 0.20 (.04) | <.001 | 1,235 (1,246) | 0.27 (.09) | .003 | 176 (324) |
| Postdoc^7^ | 0.25 (.06) | <.001 | 299 (2,481) | – | – | – | – | – | – |
| Assistant Professor^7^ | -0.13 (.07) | .079 | 201 | – | – | – | -0.33 (.10) | .001 | 201 (299) |
| Tier 2^8^ | -0.11 (.04) | .004 | 922 (1,436) | -0.11 (.04) | .012 | 763 (1,202) | -0.18 (.10) | .070 | 159 (234) |
| Tier 3^8^ | -0.12 (.04) | .010 | 623 | -0.08 (.05) | .104 | 516 | -0.31 (.12) | .010 | 107 |
| Biology^9^ | -0.02 (.05) | .718 | 784 (841) | -0.02 (.05) | .722 | 595 (712) | -0.004 (.11) | .974 | 189 (129) |
| Economics^9^ | 0.01 (.05) | .832 | 583 | 0.01 (.06) | .789 | 536 | -0.15 (.17) | .372 | 47 |
| Physics^9^ | -0.15 (.05) | .002 | 773 | -0.15 (.05) | .007 | 638 | -0.22 (.12) | .078 | 135 |
| *Note.* Referent groups *n*s given parenthetically under focal group n; reference groups are as follows: ^1^ Men, ^2^ Non-parent, ^3^ White, ^4^ heterosexual, ^5^ not first generation, ^6^ no disability, ^7^ doctoral student, ^8^ tier 1, and ^9^ psychology. | | | | | | | | | |

| Table S6. Unstandardized regression coefficients, standard errors, *p*-values, and *n*’s for change in support from mentors by socio-demographic status for the full sample, doctoral student subsample, and postdoctoral scholar and assistant professor subsample. | | | | | | | | | |
| --- | --- | --- | --- | --- | --- | --- | --- | --- | --- |
|  | Full sample  *R^2^* = .03, *df* = 15, 2,960 | | | Doctoral Students  *R^2^* = .03, *df* = 13, 2,465 | | | Postdoctoral Scholars and Assistant Professors  *R^2^* = .07, *df* = 14, 482 | | |
|  | B (SE) | *p* | *n* | B (SE) | *p* | *n* | B (SE) | *p* | *n* |
| Woman or non-binary^1^ | 0.05 (.04) | .159 | 1,526 (1,450) | 0.06 (.04) | .183 | 1,314 (1,165) | 0.04 (.08) | .645 | 212 (285) |
| Primary caregiver^2^ | -0.27 (.11) | .015 | 74 (2,642) | -0.19 (.15) | .207 | 40 (2,324) | -0.33 (.16) | .033 | 34 (318) |
| Non-primary caregiver^2^ | -0.01 (.07) | .879 | 260 | -0.06 (.09) | .522 | 115 | 0.05 (.09) | .598 | 145 |
| Asian^3^ | 0.20 (.04) | <.001 | 890 (1,583) | 0.22 (.04) | <.001 | 777 (1,269) | 0.12 (.09) | .190 | 113 (314) |
| URM^3^ | 0.18 (.05) | <.001 | 503 | 0.21 (.05) | <.001 | 433 | 0.004 (.11) | .975 | 70 |
| Sexual minority^4^ | 0.01 (.05) | .840 | 547 (2,429) | 0.01 (.05) | .776 | 492 (1,987) | -0.03 (.13) | .842 | 55 (442) |
| First generation college^5^ | -0.05 (.04) | .166 | 819 (2,157) | -0.05 (.04) | .222 | 665 (1,814) | -0.04 (.08) | .604 | 154 (343) |
| Disability^6^ | -0.13 (.04) | <.001 | 1,411 (1,565) | -0.12 (.04) | .002 | 1,236 (1,243) | -0.18 (.08) | .026 | 175 (322) |
| Postdoc^7^ | -0.06 (.06) | .302 | 297 (2,479) | – | – | – | – | – | – |
| Assistant Professor^7^ | -0.36 (.07) | <.001 | 200 | – | – | – | -0.37 (.09) | <.001 | 200 (297) |
| Tier 2^8^ | 0.10 (.04) | .009 | 922 (1,432) | 0.10 (.04) | .017 | 764 (1,200) | 0.11 (.09) | .205 | 158 (232) |
| Tier 3^8^ | 0.04 (.05) | .340 | 622 | 0.05 (.05) | .344 | 515 | 0.05 (.10) | .621 | 107 |
| Biology^9^ | -0.04 (.05) | .362 | 779 (841) | -0.02 (.05) | .686 | 592 (712) | -0.14 (.10) | .164 | 187 (129) |
| Economics^9^ | -0.20 (.05) | <.001 | 581 | -0.22 (.06) | <.001 | 534 | 0.01 (.15) | .964 | 47 |
| Physics^9^ | -0.03 (.05) | .573 | 775 | -0.02 (.06) | .686 | 641 | -0.06 (.11) | .557 | 134 |
| *Note.* Referent groups *n*s given parenthetically under focal group n; reference groups are as follows: ^1^ Men, ^2^ Non-parent, ^3^ White, ^4^ heterosexual, ^5^ not first generation, ^6^ no disability, ^7^ doctoral student, ^8^ tier 1, and ^9^ psychology. | | | | | | | | | |

| Table S7. Unstandardized regression coefficients, standard errors, *p*-values, and *n*’s for work impacts due to physical health symptoms by socio-demographic status for the full sample, doctoral student subsample, and postdoctoral scholar and assistant professor subsample. | | | | | | | | | |
| --- | --- | --- | --- | --- | --- | --- | --- | --- | --- |
|  | Full sample  *R^2^* = .17, *df* = 15, 2,803 | | | Doctoral Students  *R^2^* = .17, *df* = 13, 2,331 | | | Postdoctoral Scholars and Assistant Professors  *R^2^* = .14, *df* = 14, 459 | | |
|  | B (SE) | *p* | *n* | B (SE) | *p* | *n* | B (SE) | *p* | *n* |
| Woman or non-binary^1^ | 0.24 (.05) | <.001 | 1,535 (1,446) | 0.22 (.06) | <.001 | 1,320 (1,160) | 0.29 (.11) | .012 | 215 (286) |
| Primary caregiver^2^ | 0.29 (.15) | .056 | 76 (2,645) | 0.06 (.20) | .763 | 40 (2,325) | 0.53 (.22) | .015 | 36 (320) |
| Non-primary caregiver^2^ | -0.01 (.09) | .953 | 260 | -0.05 (.12) | .696 | 115 | 0.07 (.13) | .607 | 145 |
| Asian^3^ | 0.09 (.05) | .116 | 889 (1,588) | 0.12 (.06) | .051 | 775 (1,273) | -0.06 (.13) | .623 | 114 (315) |
| URM^3^ | 0.29 (.06) | <.001 | 504 | 0.33 (.07) | <.001 | 432 | 0.09 (.16) | .549 | 72 |
| Sexual minority^4^ | 0.09 (.06) | .166 | 548 (2,433) | 0.08 (.07) | .256 | 493 (1,987) | 0.14 (.17) | .408 | 55 (446) |
| First generation college^5^ | 0.12 (.05) | .021 | 816 (2,165) | 0.16 (.06) | .006 | 661 (1,819) | -0.07 (.12) | .536 | 155 (346) |
| Disability^6^ | 0.93 (.05) | <.001 | 1,412 (1,569) | 0.96 (.05) | <.001 | 1,235 (1,245) | 0.76 (.11) | <.001 | 177 (324) |
| Postdoc^7^ | -0.34 (.08) | <.001 | 300 (2,480) | – | – | – | – | – | – |
| Assistant Professor^7^ | -0.14 (.10) | .171 | 201 | – | – | – | 0.13 (.13) | .298 | 201 (300) |
| Tier 2^8^ | -0.03 (.05) | .621 | 922 (1,437) | -0.06 (.06) | .282 | 763 (1,202) | 0.11 (.13) | .369 | 159 (235) |
| Tier 3^8^ | 0.08 (.06) | .190 | 622 | 0.10 (.07) | .138 | 515 | -0.003 (.15) | .985 | 107 |
| Biology^9^ | 0.12 (.06) | .062 | 783 (844) | 0.11 (.07) | .123 | 594 (714) | 0.13 (.14) | .328 | 189 (130) |
| Economics^9^ | -0.01 (.07) | .891 | 579 | -0.03 (.08) | .742 | 532 | 0.07 (.21) | .733 | 47 |
| Physics^9^ | 0.03 (.07) | .644 | 775 | 0.02 (.08) | .750 | 640 | 0.02 (.15) | .910 | 135 |
| *Note.* Referent groups *n*s given parenthetically under focal group n; reference groups are as follows: ^1^ Men, ^2^ Non-parent, ^3^ White, ^4^ heterosexual, ^5^ not first generation, ^6^ no disability, ^7^ doctoral student, ^8^ tier 1, and ^9^ psychology. | | | | | | | | | |

| Table S8. Unstandardized regression coefficients, standard errors, *p*-values, and *n*’s for work impacts due to mental health symptoms by socio-demographic status for the full sample, doctoral student subsample, and postdoctoral scholar and assistant professor subsample. | | | | | | | | | |
| --- | --- | --- | --- | --- | --- | --- | --- | --- | --- |
|  | Full sample  *R^2^* = .23, *df* = 15, 2,887 | | | Doctoral Students  *R^2^* = .21, *df* = 13, 2,403 | | | Postdoctoral Scholars and Assistant Professors  *R^2^* = .23, *df* = 14, 471 | | |
|  | B (SE) | *p* | *n* | B (SE) | *p* | *n* | B (SE) | *p* | *n* |
| Woman or non-binary^1^ | 0.23 (.05) | <.001 | 1,536 (1,448) | 0.24 (.05) | <.001 | 1,321 (1,162) | 0.22 (.11) | .058 | 215 (286) |
| Primary caregiver^2^ | 0.19 (.14) | .182 | 76 (2,648) | 0.01 (.19) | .949 | 40 (2,328) | 0.37 (.22) | .090 | 36 (320) |
| Non-primary caregiver^2^ | -0.17 (.09) | .050 | 260 | -0.12 (.12) | .316 | 115 | -0.21 (.13) | .109 | 145 |
| Asian^3^ | -0.10 (.05) | .069 | 888 (1,590) | -0.08 (.06) | .156 | 774 (1,275) | -0.19 (.13) | .142 | 114 (315) |
| URM^3^ | 0.11 (.06) | .070 | 506 | 0.12 (.07) | .090 | 434 | 0.10 (.15) | .523 | 72 |
| Sexual minority^4^ | 0.26 (.06) | <.001 | 548 (2,436) | 0.24 (.06) | <.001 | 493 (1,990) | 0.38 (.18) | .030 | 55 (446) |
| First generation college^5^ | 0.04 (.05) | .467 | 818 (2,166) | 0.08 (.06) | .150 | 663 (1,820) | -0.19 (.12) | .096 | 155 (346) |
| Disability^6^ | 1.07 (.05) | <.001 | 1,415 (1,569) | 1.08 (.05) | <.001 | 1,238 (1,245) | 1.02 (.11) | <.001 | 177 (324) |
| Postdoc^7^ | -0.29 (.08) | <.001 | 300 (2,483) | – | – | – | – | – | – |
| Assistant Professor^7^ | -0.34 (.10) | <.001 | 201 | – | – | – | -0.04 (.13) | .760 | 201 (300) |
| Tier 2^8^ | -0.05 (.05) | .346 | 924 (1,436) | -0.06 (.06) | .326 | 765 (1,201) | -0.05 (.13) | .710 | 159 (235) |
| Tier 3^8^ | -0.02 (.06) | .754 | 624 | -0.002 (.06) | .969 | 517 | -0.11 (.15) | .453 | 107 |
| Biology^9^ | 0.07 (.06) | .217 | 786 (844) | 0.08 (.07) | .238 | 597 (714) | 0.04 (.14) | .767 | 189 (130) |
| Economics^9^ | 0.04 (.07) | .584 | 580 | 0.06 (.07) | .450 | 533 | -0.13 (.21) | .547 | 47 |
| Physics^9^ | 0.04 (.06) | .507 | 774 | 0.09 (.07) | .236 | 639 | -0.17 (.15) | .281 | 135 |
| *Note.* Referent groups *n*s given parenthetically under focal group n; reference groups are as follows: ^1^ Men, ^2^ Non-parent, ^3^ White, ^4^ heterosexual, ^5^ not first generation, ^6^ no disability, ^7^ doctoral student, ^8^ tier 1, and ^9^ psychology. | | | | | | | | | |

| Table S9. Unstandardized regression coefficients, standard errors, *p*-values, and *n*’s for work impacts due to additional caretaking responsibilities by socio-demographic status for the full sample, doctoral student subsample, and postdoctoral scholar and assistant professor subsample. | | | | | | | | | |
| --- | --- | --- | --- | --- | --- | --- | --- | --- | --- |
|  | Full sample  *R^2^* = .31, *df* = 15, 2,163 | | | Doctoral Students  *R^2^* = .22, *df* = 13, 1,774 | | | Postdoctoral Scholars and Assistant Professors  *R^2^* = .56, *df* = 14, 376 | | |
|  | B (SE) | *p* | *n* | B (SE) | *p* | *n* | B (SE) | *p* | *n* |
| Woman or non-binary^1^ | 0.01 (.06) | .823 | 1,535 (1,444) | -0.02 (.06) | .695 | 1,320 (1,158) | 0.16 (.12) | .198 | 215 (286) |
| Primary caregiver^2^ | 2.58 (.14) | <.001 | 76 (2,643) | 2.60 (.19) | <.001 | 40 (2,323) | 2.61 (.21) | <.001 | 36 (320) |
| Non-primary caregiver^2^ | 1.90 (.09) | <.001 | 260 | 1.71 (.12) | <.001 | 115 | 2.16 (.13) | <.001 | 145 |
| Asian^3^ | 0.24 (.06) | <.001 | 886 (1,589) | 0.30 (.07) | <.001 | 772 (1,274) | -0.07 (.14) | .602 | 114 (315) |
| URM^3^ | 0.34 (.07) | <.001 | 504 | 0.33 (.08) | <.001 | 432 | 0.41 (.18) | .021 | 72 |
| Sexual minority^4^ | -0.10 (.07) | .162 | 548 (2,431) | -0.08 (.07) | .286 | 493 (1,985) | -0.20 (.20) | .327 | 55 (446) |
| First generation college^5^ | 0.16 (.06) | .007 | 816 (2,163) | 0.17 (.06) | .008 | 661 (1,817) | 0.11 (.12) | .354 | 155 (346) |
| Disability^6^ | 0.44 (.05) | <.001 | 1,413 (1,566) | 0.45 (.06) | <.001 | 1,236 (1,242) | 0.35 (.12) | .004 | 177 (324) |
| Postdoc^7^ | -0.27 (.09) | .002 | 300 (2,478) | – | – | – | – | – | – |
| Assistant Professor^7^ | 0.14 (.11) | .177 | 201 | – | – | – | 0.28 (.13) | .033 | 201 (300) |
| Tier 2^8^ | 0.03 (.06) | .591 | 923 (1,433) | 0.06 (.07) | .369 | 764 (1,198) | -0.14 (.13) | .280 | 159 (235) |
| Tier 3^8^ | 0.01 (.07) | .915 | 623 | 0.02 (.07) | .740 | 516 | -0.08 (.15) | .602 | 107 |
| Biology^9^ | 0.12 (.07) | .098 | 785 (843) | 0.13 (.08) | .096 | 596 (713) | 0.06 (.14) | .652 | 189 (130) |
| Economics^9^ | -0.001 (.08) | .993 | 579 | -0.02 (.09) | .803 | 532 | 0.20 (.22) | .382 | 47 |
| Physics^9^ | -0.001 (.07) | .986 | 772 | 0.01 (.08) | .912 | 637 | -0.10 (.16) | .542 | 135 |
| *Note.* Referent groups *n*s given parenthetically under focal group n; reference groups are as follows: ^1^ Men, ^2^ Non-parent, ^3^ White, ^4^ heterosexual, ^5^ not first generation, ^6^ no disability, ^7^ doctoral student, ^8^ tier 1, and ^9^ psychology. | | | | | | | | | |

**Supplemental Multilevel Analyses**

Preliminary multilevel analyses indicated no need to account for the effect of participants being in the same department (i.e., participants’ department did not account for a significant amount of the variance in any of the COVID-19 impacts; see supplement); thus, we present the non-nested regression models in the main text. The results of the multilevel analyses can be found in tables S3 and S4.

| Table S10. Unstandardized regression coefficients, standard errors, and p-values for work impacts, produced by multilevel mixed-effects regression models. | | | | | | | | | | | | | | | |
| --- | --- | --- | --- | --- | --- | --- | --- | --- | --- | --- | --- | --- | --- | --- | --- |
|  | Research Progress | | |  | Workload | | |  | Concern about career advancement | | |  | Support from mentors | | |
|  | Coef. | SE | *p* |  | Coef. | SE | *p* |  | Coef. | SE | *p* |  | Coef. | SE | *p* |
| Woman or non-binary^1^ | -0.02 | 0.05 | .719 |  | 0.14 | 0.04 | .001 |  | 0.01 | 0.04 | .805 |  | 0.06 | 0.04 | .160 |
| Primary caregiver^2^ | 0.47 | 0.17 | .006 |  | 0.22 | 0.13 | .097 |  | -0.22 | 0.14 | .107 |  | 0.22 | 0.14 | .115 |
| Non-primary caregiver^2^ | 0.74 | 0.15 | <.001 |  | 0.23 | 0.12 | .061 |  | -0.29 | 0.12 | .018 |  | 0.19 | 0.13 | .144 |
| Asian^3^ | -0.004 | 0.05 | .944 |  | -0.08 | 0.04 | .067 |  | 0.06 | 0.04 | .146 |  | 0.20 | 0.05 | <.001 |
| URM^3^ | 0.02 | 0.06 | .707 |  | 0.07 | 0.05 | .145 |  | 0.11 | 0.05 | .040 |  | 0.20 | 0.05 | <.001 |
| Sexual minority^4^ | -0.03 | 0.06 | .665 |  | -0.04 | 0.05 | .415 |  | 0.06 | 0.05 | .248 |  | 0.01 | 0.05 | .812 |
| First generation college^5^ | -0.01 | 0.05 | .908 |  | 0.05 | 0.04 | .250 |  | -0.02 | 0.04 | .659 |  | -0.07 | 0.04 | .103 |
| Disability^6^ | -0.23 | 0.05 | <.001 |  | 0.13 | 0.04 | .001 |  | 0.22 | 0.04 | <.001 |  | -0.10 | 0.04 | .009 |
| Postdoc^7^ | -0.05 | 0.08 | .539 |  | -0.11 | 0.07 | .107 |  | 0.34 | 0.07 | <.001 |  | -0.07 | 0.07 | .343 |
| Assistant Professor^7^ | -0.21 | 0.10 | .045 |  | 0.46 | 0.08 | <.001 |  | -0.19 | 0.08 | .021 |  | -0.36 | 0.08 | <.001 |
| Tercile 2^8^ | 0.11 | 0.06 | .043 |  | 0.08 | 0.04 | .078 |  | -0.11 | 0.05 | .019 |  | 0.12 | 0.05 | .011 |
| Tercile 3^8^ | 0.14 | 0.06 | .028 |  | 0.17 | 0.05 | <.001 |  | -0.10 | 0.05 | .061 |  | 0.07 | 0.05 | .175 |
| Biology^9^ | -0.20 | 0.06 | .003 |  | -0.10 | 0.05 | .050 |  | -0.05 | 0.05 | .387 |  | -0.03 | 0.05 | .595 |
| Economics^9^ | 0.14 | 0.07 | .060 |  | 0.07 | 0.06 | .220 |  | -0.01 | 0.06 | .856 |  | -0.15 | 0.06 | .016 |
| Physics^9^ | -0.05 | 0.07 | .493 |  | -0.13 | 0.05 | .014 |  | -0.19 | 0.06 | .001 |  | 0.02 | 0.06 | .713 |
| *Note.* Referent groups are as follows: ^1^ men, ^2^ Non-parent, ^3^ White, ^4^ heterosexual, ^5^ not first generation, ^6^ no disability, ^7^ doctoral student, ^8^ tier 1, and ^9^ psychology. Multilevel generalized mixed-effects models (MLM=persons embedded in departments) were run using the GLLAMM command in Stata. | | | | | | | | | | | | | | | |

| Table S11. Unstandardized regression coefficients, standard errors, and p-values for work disruptions, produced by multilevel mixed-effects regression models. | | | | | | | | | | | |
| --- | --- | --- | --- | --- | --- | --- | --- | --- | --- | --- | --- |
|  | Physical health symptoms | | |  | Mental health symptoms | | |  | Additional caretaking responsibilities | | |
|  | Coef. | SE | *p* |  | Coef. | SE | *p* |  | Coef. | SE | *p* |
| Woman or non-binary^1^ | 0.23 | 0.06 | <.001 |  | 0.20 | 0.05 | <.001 |  | -0.02 | 0.06 | .755 |
| Primary caregiver^2^ | -0.11 | 0.19 | .567 |  | -0.23 | 0.18 | .210 |  | -0.56 | 0.18 | .002 |
| Non-primary caregiver^2^ | -0.08 | 0.17 | .650 |  | -0.07 | 0.16 | .669 |  | -2.61 | 0.16 | <.001 |
| Asian^3^ | 0.10 | 0.06 | .112 |  | -0.12 | 0.06 | .034 |  | 0.24 | 0.07 | <.001 |
| URM^3^ | 0.32 | 0.07 | <.001 |  | 0.13 | 0.07 | .050 |  | 0.32 | 0.08 | <.001 |
| Sexual minority^4^ | 0.11 | 0.07 | .107 |  | 0.24 | 0.06 | <.001 |  | -0.13 | 0.07 | .087 |
| First generation college^5^ | 0.12 | 0.06 | .047 |  | 0.05 | 0.06 | .336 |  | 0.19 | 0.06 | .003 |
| Disability^6^ | 0.99 | 0.05 | <.001 |  | 1.10 | 0.05 | <.001 |  | 0.43 | 0.06 | <.001 |
| Postdoc^7^ | -0.31 | 0.09 | .001 |  | -0.25 | 0.09 | .005 |  | -0.33 | 0.10 | .001 |
| Assistant Professor^7^ | -0.03 | 0.12 | .785 |  | -0.34 | 0.11 | .002 |  | 0.09 | 0.12 | .445 |
| Tercile 2^8^ | -0.09 | 0.06 | .123 |  | -0.10 | 0.06 | .101 |  | 0.03 | 0.06 | .690 |
| Tercile 3^8^ | 0.02 | 0.07 | .738 |  | -0.05 | 0.07 | .419 |  | -0.03 | 0.07 | .701 |
| Biology^9^ | 0.11 | 0.07 | .107 |  | 0.05 | 0.07 | .490 |  | 0.15 | 0.08 | .045 |
| Economics^9^ | -0.03 | 0.08 | .686 |  | -0.01 | 0.08 | .886 |  | 0.03 | 0.09 | .774 |
| Physics^9^ | -0.04 | 0.07 | .570 |  | -0.03 | 0.07 | .730 |  | 0.01 | 0.08 | .891 |
| *Note.* Referent groups are as follows: ^1^ men, ^2^ Non-parent, ^3^ White, ^4^ heterosexual, ^5^ not first generation, ^6^ no disability, ^7^ doctoral student, ^8^ tier 1, and ^9^ psychology. Multilevel generalized mixed-effects models (MLM=persons embedded in departments) were run using the GLLAMM command in Stata. | | | | | | | | | | | |

**Supplemental Workplace Outcome Measures**

To examine the negative impact that the COVID-19 pandemic is having on academic scientists generally, we conducted five separate multiple regressions with the seven COVID-19 outcomes predicting job satisfaction, professional role confidence, turnover intentions, burnout disengagement, and burnout exhaustion (tables S5- S9). For all measures, we calculated the mean score for each participant.

**Job Satisfaction.** To measure overall job satisfaction, we used the 10-item Job in General subscale of the Job Descriptive Index, which we adapted to be relevant to the academic environment, and one item we added on mentoring (α = .87) [4]. Participants were asked to rate their overall satisfaction on different aspects of their academic work on a 5-point Likert scale (1 = *Extremely dissatisfied* to 5 = *Extremely satisfied*). Example items include “Amount of variety in my work,” and “Amount of responsibility.” The original item we added asked about satisfaction regarding “Relationship with mentors.”

**Professional role confidence.** We used 6 items to assess participants’ confidence in their ability to be successful in their academic field, which we adapted from by Cech et al. [5] to reflect the academic environment (α = .86). Participants responded to items including “I will advance to the next stage in my career,” and “My current field is the right one for me” on a 4-point Likert scale ranging from 1 = *Not at all confident* to 4 = *Very confident.*

**Turnover intentions.** Turnover intention was measured with four items from the organizational withdrawal scale (α = .70) [6]. Participants responded to the frequency with which they had “Completed work or school assignments late” and “Thought about quitting because of school or work-related issues” within the past year on a 5-point Likert scale (1 = *Never* to 5 = *Once a week or more*).

**Burnout disengagement and burnout exhaustion.** *Burnout disengagement* (α = .69) was measured by adapting three items from the burnout disengagement subscale developed by Demerouti et al.^7^ The items asked about the frequency that participants “Talked about your work in a negative way” and “Done your work without thinking, almost mechanically” on a 5-point Likert scale (1 = *Never* to 5 = *Once a week or more*). *Burnout exhaustion* (α = .78) was measured using 4 items from the burnout exhaustion subscale developed by Demerouti et al [7]. Participants responded to the degree to which they agree or disagree with statements such as “After work, I tend to need a lot of time to relax and feel better” and “I can tolerate the pressure of my work well” on a 5-point Likert scale (1 = *Strongly disagree* to 5 = *Strongly agree*).

**Supplemental Workplace Outcome Results**

**Job Satisfaction (Table S12)**. Across all subsamples, decreased workload, decreased concern about career advancement, increased mentor support, and experiencing fewer work disruptions associated with mental health symptoms significantly predicted greater job satisfaction.

Increased research progress and fewer work disruptions due to physical health symptoms significantly predicted greater job satisfaction in the full sample and the doctoral student subsample. While additional caretaking responsibilities was not significantly associated with job satisfaction in the full sample or the postdoctoral scholar and assistant professor sample, increased caretaking responsibilities was significantly associated with lower job satisfaction in the doctoral student subsample.

| Table S12. Unstandardized regression coefficients, standard errors, and p-values for COVID-19 impacts on job satisfaction for the full sample, doctoral student subsample, and postdoctoral scholar and assistant professor subsample. | | | | | | | | | | | |
| --- | --- | --- | --- | --- | --- | --- | --- | --- | --- | --- | --- |
|  | Full sample  *R^2^* = .17, *df* = 7, 2,266 | | |  | Doctoral Students  *R^2^* = .17, *df* = 7, 1,855 | | |  | Postdoc and Asst Prof  *R^2^* = .19, *df* = 7, 403 | | |
|  | Coef. | SE | *p* |  | Coef. | SE | *p* |  | Coef. | SE | *p* |
| Change in research progress | 0.04 | 0.01 | .001 |  | 0.05 | 0.01 | .001 |  | 0.06 | 0.03 | .048 |
| Change in workload | -0.07 | 0.02 | <.001 |  | -0.07 | 0.02 | <.001 |  | -0.11 | 0.03 | .002 |
| Change in concern about career advancement | -0.04 | 0.01 | .002 |  | -0.04 | 0.02 | .022 |  | -0.09 | 0.03 | .005 |
| Change in mentor support | 0.15 | 0.01 | <.001 |  | 0.17 | 0.02 | <.001 |  | 0.13 | 0.04 | <.001 |
| Work disruptions due to physical health symptoms | -0.07 | 0.01 | <.001 |  | -0.07 | 0.01 | <.001 |  | -0.03 | 0.03 | .295 |
| Work disruptions due to mental health symptoms | -0.08 | 0.01 | <.001 |  | -0.06 | 0.01 | <.001 |  | -0.10 | 0.03 | .001 |
| Work disruptions due to additional caretaking responsibilities | -0.01 | 0.01 | .145 |  | -0.05 | 0.01 | <.001 |  | 0.03 | 0.02 | .090 |

**Professional role confidence (Table S13).**  Decreased concern about career advancement, increased mentor support, and fewer work disruptions from mental health symptoms predicted higher professional role confidence across all samples.

There were also notable differences between the subsamples such that both increased research progress and workload were positively associated with professional role confidence in the full sample and doctoral student subsample but not in the postdoctoral scholar and assistant professor subsample. Furthermore, fewer work disruptions due to physical health symptoms significantly predicted increased professional role confidence in the full sample only. Finally, greater work disruptions due to additional caretaking responsibilities significantly predicted professional role confidence in the postdoctoral scholar and assistant professor subsample only.

| Table S13. Unstandardized regression coefficients, standard errors, and p-values for COVID-19 impacts on professional role confidence for the full sample, doctoral student subsample, and postdoctoral scholar and assistant professor subsample. | | | | | | | | | | | |
| --- | --- | --- | --- | --- | --- | --- | --- | --- | --- | --- | --- |
|  | Full sample  *R^2^* = .12, *df* = 7, 2,262 | | |  | Doctoral Students  *R^2^* = .12, *df* = 7, 1,852 | | |  | Postdoc and Asst Prof  *R^2^* = .15, *df* = 7, 402 | | |
|  | Coef. | SE | *p* |  | Coef. | SE | *p* |  | Coef. | SE | *p* |
| Change in research progress | 0.06 | 0.02 | <.001 |  | 0.06 | 0.02 | <.001 |  | 0.07 | 0.04 | .054 |
| Change in workload | 0.08 | 0.02 | <.001 |  | 0.09 | 0.02 | <.001 |  | 0.06 | 0.04 | .128 |
| Change in concern about career advancement | -0.11 | 0.02 | <.001 |  | -0.11 | 0.02 | <.001 |  | -0.11 | 0.04 | .004 |
| Change in mentor support | 0.08 | 0.02 | <.001 |  | 0.08 | 0.02 | <.001 |  | 0.10 | 0.04 | .023 |
| Work disruptions due to physical health symptoms | -0.05 | 0.02 | 0.001 |  | -0.05 | 0.02 | .007 |  | -0.06 | 0.04 | .090 |
| Work disruptions due to mental health symptoms | -0.09 | 0.01 | <.001 |  | -0.09 | 0.02 | <.001 |  | -0.10 | 0.03 | .006 |
| Work disruptions due to additional caretaking responsibilities | 0.02 | 0.01 | .183 |  | -0.004 | 0.01 | .796 |  | 0.08 | 0.02 | .001 |

**Turnover intentions (Table S14).** Across samples, only increased work disruptions due to mental health symptoms predicted more turnover intentions. Workload and additional caretaking responsibilities were not significantly associated with turnover intentions across all samples.

Whereas decreased research progress was significantly associated with turnover intentions for the full sample, it was nonsignificant in the two separate subsamples. Increased concern about career advancement was significantly associated with higher turnover intentions in the full sample and postdoctoral researcher and assistant professor subsample but not the doctoral student subsample. Further, decreased mentor support and increased work disruptions due to physical health symptoms significantly predicted higher turnover intentions in the full sample and doctoral student subsample, but not in the postdoctoral scholar and assistant professor subsample.

| Table S14. Unstandardized regression coefficients, standard errors, and p-values for COVID-19 impacts on turnover intentions for the full sample, doctoral student subsample, and postdoctoral scholar and assistant professor subsample. | | | | | | | | | | | |
| --- | --- | --- | --- | --- | --- | --- | --- | --- | --- | --- | --- |
|  | Full sample  *R^2^* = .20, *df* = 7, 2,265 | | |  | Doctoral Students  *R^2^* = .20, *df* = 7, 1,855 | | |  | Postdoc and Asst Prof  *R^2^* = .22, *df* = 7, 402 | | |
|  | Coef. | SE | *p* |  | Coef. | SE | *p* |  | Coef. | SE | *p* |
| Change in research progress | -0.03 | 0.02 | .044 |  | -0.03 | 0.02 | .137 |  | -0.05 | 0.04 | .245 |
| Change in workload | -0.02 | 0.02 | .238 |  | -0.04 | 0.02 | .058 |  | 0.04 | 0.05 | .338 |
| Change in concern about career advancement | 0.04 | 0.02 | .037 |  | 0.03 | 0.02 | .227 |  | 0.10 | 0.04 | .023 |
| Change in mentor support | -0.06 | 0.02 | .001 |  | -0.06 | 0.02 | .006 |  | -0.08 | 0.05 | .086 |
| Work disruptions due to physical health symptoms | 0.05 | 0.02 | .003 |  | 0.07 | 0.02 | <.001 |  | -0.04 | 0.04 | .307 |
| Work disruptions due to mental health symptoms | 0.25 | 0.02 | <.001 |  | 0.25 | 0.02 | <.001 |  | 0.29 | 0.04 | <.001 |
| Work disruptions due to additional caretaking responsibilities | -0.001 | 0.01 | .910 |  | -0.01 | 0.02 | .742 |  | -0.004 | 0.03 | .870 |

**Burnout exhaustion (Table S15).** Across all samples, increased workload, concern about career advancement, and work disruptions due to mental health symptoms predicted greater burnout exhaustion. Research progress was not significantly associated with burnout exhaustion across all samples.

There were notable differences across the subsamples. Decreased mentor support and increased work disruptions due to physical health symptoms were significantly associated with burnout exhaustion in the full sample and doctoral student subsample but not in the postdoctoral scholar and assistant professor subsample. Increased work disruptions due to additional caretaking responsibilities was significantly associated with decreased burnout exhaustion in the full sample only.

| Table S15. Unstandardized regression coefficients, standard errors, and p-values for COVID-19 impacts on burnout exhaustion for the full sample, doctoral student subsample, and postdoctoral scholar and assistant professor subsample. | | | | | | | | | | | |
| --- | --- | --- | --- | --- | --- | --- | --- | --- | --- | --- | --- |
|  | Full sample  *R^2^* = .26, *df* = 7, 2,267 | | |  | Doctoral Students  *R^2^* = .26, *df* = 7, 1,856 | | |  | Postdoc and Asst Prof  *R^2^* = .25, *df* = 7, 403 | | |
|  | Coef. | SE | *p* |  | Coef. | SE | *p* |  | Coef. | SE | *p* |
| Change in research progress | -0.02 | 0.01 | .219 |  | -0.02 | 0.02 | .155 |  | -0.03 | 0.04 | .509 |
| Change in workload | 0.10 | 0.02 | <.001 |  | 0.10 | 0.02 | <.001 |  | 0.13 | 0.04 | .004 |
| Change in concern about career advancement | 0.06 | 0.02 | <.001 |  | 0.06 | 0.02 | <.001 |  | 0.08 | 0.04 | .041 |
| Change in mentor support | -0.09 | 0.02 | <.001 |  | -0.09 | 0.02 | <.001 |  | -0.10 | 0.04 | .026 |
| Work disruptions due to physical health symptoms | 0.08 | 0.01 | <.001 |  | 0.08 | 0.02 | <.001 |  | 0.03 | 0.04 | .442 |
| Work disruptions due to mental health symptoms | 0.22 | 0.01 | <.001 |  | 0.21 | 0.02 | <.001 |  | 0.25 | 0.04 | <.001 |
| Work disruptions due to additional caretaking responsibilities | -0.02 | 0.01 | .038 |  | -0.01 | 0.01 | .417 |  | -0.03 | 0.02 | .276 |

**Burnout disengagement (Table S16).** Increased workload, increased concern about career advancement, decreased mentor support, and increased work disruptions due to mental health symptoms significantly predicted increased burnout disengagement across all samples. Work disruptions due to physical health symptoms were not associated with burnout disengagement across all samples.

Surprisingly, increased work disruptions due to additional caretaking responsibilities significantly predicted lower burnout disengagement in the full sample and doctoral student subsample, and was not associated with burnout disengagement in the postdoctoral scholar and assistant professor subsample. Although research progress was not significantly associated with burnout disengagement in the full sample or doctoral student subsample, decreased research progress was significantly associated with increased burnout disengagement in the postdoctoral scholar and assistant professor subsample.

| Table S16. Unstandardized regression coefficients, standard errors, and p-values for COVID-19 impacts on burnout disengagement for the full sample, doctoral student subsample, and postdoctoral scholar and assistant professor subsample. | | | | | | | | | | | |
| --- | --- | --- | --- | --- | --- | --- | --- | --- | --- | --- | --- |
|  | Full sample  *R^2^* = .19, *df* = 7, 2,265 | | |  | Doctoral Students  *R^2^* = .17, *df* = 7, 1,855 | | |  | Postdoc and Asst Prof  *R^2^* = .25, *df* = 7, 403 | | |
|  | Coef. | SE | *p* |  | Coef. | SE | *p* |  | Coef. | SE | *p* |
| Change in research progress | -0.04 | 0.02 | .060 |  | -0.03 | 0.02 | .176 |  | -0.10 | 0.05 | .036 |
| Change in workload | 0.06 | 0.02 | .006 |  | 0.05 | 0.03 | .041 |  | 0.11 | 0.05 | .043 |
| Change in concern about career advancement | 0.07 | 0.02 | .003 |  | 0.06 | 0.02 | .020 |  | 0.11 | 0.05 | .023 |
| Change in mentor support | -0.14 | 0.02 | <.001 |  | -0.13 | 0.02 | <.001 |  | -0.21 | 0.06 | <.001 |
| Work disruptions due to physical health symptoms | 0.02 | 0.02 | .300 |  | 0.02 | 0.02 | .265 |  | -0.03 | 0.05 | .517 |
| Work disruptions due to mental health symptoms | 0.28 | 0.02 | <.001 |  | 0.26 | 0.02 | <.001 |  | 0.33 | 0.05 | <.001 |
| Work disruptions due to additional caretaking responsibilities | -0.06 | 0.02 | <.001 |  | -0.06 | 0.02 | .001 |  | -0.04 | 0.03 | .192 |

**Supplemental Correlations**

| Table S17. Means, standard deviations, and correlations of COVID-19 impact variables for the full sample, doctoral student subsample, and postdoctoral scholar and assistant professor subsample. | | | | | | | | | |
| --- | --- | --- | --- | --- | --- | --- | --- | --- | --- |
|  |  |  |  |  |  |  |  | Doc subsample/  Postdoc & Asst Prof subsample | |
| Variable | 1 | 2 | 3 | 4 | 5 | 6 | 7 | *M* | *SD* |
| 1. Research progress |  | .17**/ -.004 | -.16**/ -.21** | .24**/ .22** | -.18**/ -.27** | -.26**/ -.26** | -.13**/ -.27** | 2.6/ 2.4 | 1.1/ 1.0 |
| 2. Workload | .14** |  | .11**/ .05 | .05*/ -.16** | .15**/ .07 | .10**/  .003 | .07*/ .19** | 3.3/ 3.4 | 0.9/ 0.9 |
| 3. Concern about career advancement | -.17** | .10** |  | -.10**/ -.10* | .21**/ .20** | .23**/ .29** | .10**/ .08 | 3.8/ 3.9 | 0.9/ 1.0 |
| 4. Support from mentor(s) | .24** | .02 | -.10** |  | -.02/ -.14** | -.08**/ -.09* | -.03/ -.14* | 2.9/ 2.7 | 1.0/ 0.8 |
| 5. Physical health symptoms | -.19** | .14** | .20** | -.03 |  | .61**/ .61** | .38**/ .27** | 2.7/ 2.4 | 1.3/ 1.2 |
| 6. Mental health symptoms | -.25** | .08** | .24** | -.07** | .61** |  | .30**/ .15* | 3.4/ 2.9 | 1.3/ 1.3 |
| 7. Additional caretaking responsibilities | -.17** | .10** | .10** | -.06* | .33** | .24** |  | 2.1/ 2.7 | 1.3/ 1.6 |
| Full sample: *M* | 2.5 | 3.3 | 3.8 | 2.9 | 2.7 | 3.3 | 2.2 |  |  |
| Full sample: *SD* | 1.1 | 0.9 | 0.9 | 0.9 | 1.3 | 1.3 | 1.4 |  |  |

*Note*. * indicates *p* < .05. ** indicates *p* < .001.

**Supplemental References**

1. A Data-Based Assessment of Research-Doctorate Programs in the United States. 2011. Available from: https://www.nationalacademies.org/our-work/an-assessment-of-research-doctorate-programs

2. Ghavami N, & Peplau LA. An intersectional analysis of gender and ethnic stereotypes: Testing three hypotheses. Psychology of Women Quarterly. 2013; 37(1):113-127.

3. Chou RS, & Feagin JR. Myth of the model minority: Asian Americans facing racism. Paradigm Press. 2015.

4. Hulin CL, Kendall L, & Smith P. The measurement of satisfaction in work and retirement. Chicago, Rand Mcnally. 1969.

5. Cech E, Rubineau B, Silbey S, & Seron C. Professional role confidence and gendered persistence in engineering. American Sociological Review*.* 2011; 76(5):641-666. Available from: http://doi.org/10.1177/0003122411420815

6. Hanisch KA, & Hulin CL. Job attitudes and organizational withdrawal: An examination of retirement and other voluntary withdrawal behaviors. Journal of Vocational Behavior. 1990; 37(1):60-78. Available from: https://doi.org/10.1016/0001-8791(90)90007-O

7. Demerouti E, Mostert K, & Bakker A.B. Burnout and work engagement: A thorough investigation of the independency of both constructs. Journal of Occupational Health Psychology. 2010; 15(3):209-222. Available from: https://doi.org/10.1037/a0019408
