# Supplementary material for: Disproportionate impacts of COVID-19 on marginalized and minoritized early-career academic scientists
Source: PLoS One. 2022 Sep 13;17(9):e0274278. doi: 10.1371/journal.pone.0274278 (PMC9469975; doi:10.1371/journal.pone.0274278)
Supplement: Attachment — Submitted filename: Response to Reviewers.docx [file pone.0274278.s002.docx]

### Response to Reviewers

*The reviewers felt this was an important piece of work that can inform policy development in higher education to mitigate the inequitable effects of the COVID-19 pandemic. Reviewer 2 offers an important critique of the analysis and reporting of the findings from postdoc and faculty sub-samples, given that they were much smaller than that of graduate students, which I ask you to particularly address. I look forward to receiving a revised version of this work.*

When submitting your revision, we need you to address these additional requirements.

1. Please ensure that your manuscript meets PLOS ONE's style requirements, including those for file naming. The PLOS ONE style templates can be found at

<https://journals.plos.org/plosone/s/file?id=wjVg/PLOSOne_formatting_sample_main_body.pdf> and <https://journals.plos.org/plosone/s/file?id=ba62/PLOSOne_formatting_sample_title_authors_affiliations.pdf>.

2. Please include your tables as part of your main manuscript and remove the individual files. Please note that supplementary tables (should remain/ be uploaded) as separate "supporting information" files.

**The tables have been added to the main text.**

3. Please provide additional details regarding participant consent. In the ethics statement in the Methods and online submission information, please ensure that you have specified what type you obtained (for instance, written or verbal, and if verbal, how it was documented and witnessed). If your study included minors, state whether you obtained consent from parents or guardians. If the need for consent was waived by the ethics committee, please include this information.

Once you have amended this/these statement(s) in the Methods section of the manuscript, please add the same text to the “Ethics Statement” field of the submission form (via “Edit Submission”).

For additional information about PLOS ONE ethical requirements for human subjects research, please refer to <http://journals.plos.org/plosone/s/submission-guidelines#loc-human-subjects-research>.

**More specific information regarding participant consent was added to the main text (lines 122-124) and the Ethics Statement was updated.**

4. In your Data Availability statement, you have not specified where the minimal data set underlying the results described in your manuscript can be found. PLOS defines a study's minimal data set as the underlying data used to reach the conclusions drawn in the manuscript and any additional data required to replicate the reported study findings in their entirety. All PLOS journals require that the minimal data set be made fully available. For more information about our data policy, please see <http://journals.plos.org/plosone/s/data-availability>.

Upon re-submitting your revised manuscript, please upload your study’s minimal underlying data set as either Supporting Information files or to a stable, public repository and include the relevant URLs, DOIs, or accession numbers within your revised cover letter. For a list of acceptable repositories, please see <http://journals.plos.org/plosone/s/data-availability#loc-recommended-repositories>. Any potentially identifying patient information must be fully anonymized.

Important: If there are ethical or legal restrictions to sharing your data publicly, please explain these restrictions in detail. Please see our guidelines for more information on what we consider unacceptable restrictions to publicly sharing data: <http://journals.plos.org/plosone/s/data-availability#loc-unacceptable-data-access-restrictions>. Note that it is not acceptable for the authors to be the sole named individuals responsible for ensuring data access.

We will update your Data Availability statement to reflect the information you provide in your cover letter.

**We have uploaded the dataset to openICPSR. We excluded demographic information to protect participant confidentiality.**

5. Please include captions for your Supporting Information files at the end of your manuscript, and update any in-text citations to match accordingly. Please see our Supporting Information guidelines for more information: <http://journals.plos.org/plosone/s/supporting-information>.

Reviewers' comments:

### Reviewer's Responses to Questions

**Comments to the Author**

1. Is the manuscript technically sound, and do the data support the conclusions?

The manuscript must describe a technically sound piece of scientific research with data that supports the conclusions. Experiments must have been conducted rigorously, with appropriate controls, replication, and sample sizes. The conclusions must be drawn appropriately based on the data presented.

Reviewer #1: Yes

Reviewer #2: Partly

2. Has the statistical analysis been performed appropriately and rigorously?

Reviewer #1: Yes

Reviewer #2: No

3. Have the authors made all data underlying the findings in their manuscript fully available?

The [PLOS Data policy](http://www.plosone.org/static/policies.action#sharing) requires authors to make all data underlying the findings described in their manuscript fully available without restriction, with rare exception (please refer to the Data Availability Statement in the manuscript PDF file). The data should be provided as part of the manuscript or its supporting information, or deposited to a public repository. For example, in addition to summary statistics, the data points behind means, medians and variance measures should be available. If there are restrictions on publicly sharing data—e.g. participant privacy or use of data from a third party—those must be specified.

Reviewer #1: Yes

Reviewer #2: No

4. Is the manuscript presented in an intelligible fashion and written in standard English?

PLOS ONE does not copyedit accepted manuscripts, so the language in submitted articles must be clear, correct, and unambiguous. Any typographical or grammatical errors should be corrected at revision, so please note any specific errors here.

Reviewer #1: Yes

Reviewer #2: Yes

### Review Comments to the Author

Please use the space provided to explain your answers to the questions above. You may also include additional comments for the author, including concerns about dual publication, research ethics, or publication ethics. (Please upload your review as an attachment if it exceeds 20,000 characters)

### Reviewer #1:

This is a well-executed and well-written assessment of the varied impact of the COVID-19 pandemic on early-career individuals in STEM. The sample sizes are adequate for most groups except for the small number of individuals who self-identify as non-binary. However, the authors note that these individuals tended to follow similar trends as women and were able to combine them with this group. While these findings are unable to describe the intersectionality will which often exacerbate many of these socio-demographic differences, this work is a great first step in understanding how this pandemic will disproportionately impact those groups that are already historically marginalized or excluded from the STEM fields and academia. These data are necessary for university to enact policy that specifically aids these groups in recovery from this pandemic in order to not further advance the minimization of these groups in STEM/academia. I strongly encourage publication of this work.

**Thank you for your review and comments of our work!**

### Reviewer #2:

This is a timely study examining the effects of COVID on the academic progress of early career scientists. While in general I think the survey methods are clear the authors do need to provide more information concerning when and how the survey was distributed as well as including a copy of the survey itself in the supplemental. Otherwise the data are not reproducible. I also do have some major concerns about the statistical analyses. Based on Supplemental Tables 1 and 2, the authors do not have enough faculty or postdoc respondents to examine the effects of race, gender, disability, or parental status on response to COVID for those two career stages. Given the high number of graduate student respondents, this paper would be much stronger if the authors only used the graduate student data for all responses except for the comparisons across career stage. Since assistant professors make up less than 10% of the responses, and postdocs make up less than 20% of the responses, they have no impact on the results for broad demographic groups. The reported results are really just the results for graduate student participants.

**Thank you for your review of our manuscript. To address the main concern regarding the relatively small sample size of postdocs and assistant professor compared to doctoral students, we replicated the 7 multiple regressions on two different subsamples (7 models exploring COVID-19 impacts for doctoral students alone and 7 models analyzing COVID-19 impacts for postdocs and assistant professors combined). In the results we present the consistent findings across all models as well as describe where results differ across subsamples. These analyses demonstrated that many differences were visible across subsamples, whereas others emerged only for some subsamples. Notably, there were cases where findings emerged only for the postdoc/assistant professor subsample despite their smaller numbers. As our overall findings were not simply a reflection of graduate students’ experiences, we choose to retain our focus on early-career researchers generally, rather than only focusing on doctoral students. This provides a more holistic understanding of negative effects of COVID-19 on academics across early career stages. With the updated presentation of results and discussion in our resubmission, we are able to show some of the universal experiences of this pandemic while also highlighting unique impacts across career stages.**

This overwhelming difference in number of responses from graduate students relative to postdocs and faculty has downstream effects on the validity of the authors’ interpretation of their results, some of which I mention in my line comments below.

Line Comments:

Line 40: “other work dimensions” is vague. Please be more specific.

**We’ve specified other work dimensions as increased workload and decreased research progress (LINE 50).**

Lines 56-63: My understanding is that most of these studies have focused on faculty, not graduate students or postdocs. Since the authors specifically query graduate students and postdocs in this study, I suggest the authors mention the career stage targeted by the studies mentioned here, as individuals at different career stages may have different experiences.

**We have added the career stage of the studies cited in the introduction. When the cited studies surveyed people in academia but did not specify career stage, we referred to the sample as “academics.” (LINES 69 – 72)**

An additional, more minor point here is that these cited studies primarily focus on effects of caregiving. There are virtually no studies examining effects of gender on response to COVID in the absence of caregiving. In other words, there are no studies comparing the effects of COVID on single women, men, and non-binary individuals without children in academia. This is an important distinction, because “woman” and “caregiver” are often conflated. Since the authors attempt to tease this apart in their own work, it is worth pointing out that the previous studies have not.

**Thank you for this observation about the state of the research. In our introduction, we have cited studies that do look at gender and caregiving separately and jointly (e.g., Myers et al. - Nature Human Behavior; Deryugina et al, 2021).**

Line 99: I suggest including the specific year this study was conducted instead of stating “the past year”, as it will be read in future years. Please also include the months this survey was live.

**We’ve added the time frame and the months the survey was open (LINES 111 AND 119).**

Since this is largely a study of graduate students (10x as many graduate student respondents as professors) I suggest the authors emphasize their survey of graduate students.

**Since we have included the additional analyses by subgroup, we have emphasized the differences and similarities across career stages throughout the document.**

Line 112: What do the authors mean by “prestige”? Please define or use a different term. Based on the supplemental material, it looks like they mean R1, R2, R3. This is not a definition of prestige, this is instead Carnegie Classification of Institutes of Higher Education.

**We defined prestige by the National Research Council rankings of PhD granting institutions; we added this detail to the main text (LINE 159). First, we got the ranked list of universities for each of the four departments and divided them into terciles (our ranking definition). We randomly selected 10 universities from each tercile. This more detailed description of the prestige variable is found in the supplement (LINES 5-8).**

Line 114: Please include IRB protocol numbers

**The IRB protocol numbers have been added to the method (LINE 126).**

Line 120: Please include justification for aggregating non-binary with woman.

**The justification for aggregating these groups was added to the method (LINES 143-148).**

For Figures 1 and 2: I suggest the authors put the sample size per bar above/next to each bar. Otherwise the visualization is a false equivalency because it implies even sample sizes across categories.

**Thank you for this suggestion, we agree that the subgroup sample size is important. We decided to add that information to the summary of results tables instead of the figures so as to retain the readability of the figures (which already contain the mean and standard error).**

Lines 281-290: This result could be largely due to the differential experiences of female graduate students, postdocs, and faculty, since the respondents are overwhelmingly graduate students. This illustrates my earlier point that it is extremely important to address the fact that faculty and postdoc respondents only make up just over 20% of the total respondents, so their voices are not heard in any of the analyses that analyze graduate students, postdocs, and faculty in aggregate.

**After re-running the analyses in the postdoc and assistant professor sample, we found that advanced early-career women did not significantly differ from men in any of the work impacts where there weren’t also differences in the doctoral student sample.**

Line 304: This statement would be stronger if the authors compared the proportion of respondents that have children by career stage (% grad students vs % postdocs vs % assistant professors)

**Thank you for this suggestion; we’ve added those details (LINE 469-470).**

Line 307: I suggest removing the quotes around “mental health crisis” here, as that might indicate to readers that the authors don’t believe graduate students are having a mental health crisis (which I don’t think is case).

**Thank you for this suggestion; we’ve removed the quotes.**

Line 319: Here the authors comment on the timing of their survey, but since they did not include the specific dates of the survey in their methods section this statement is ungrounded. Please add the year and months this survey was live in the methods, and re-state the time frame of the survey here, so the readers do not need to flip back to the methods section to find it.

**This has been added to the limitations section (LINE 495).**

Lines 335: Please include the small sample size of assistant professors and postdocs, as well as indicating why the study did not speak to intersectionality (low sample size for each category).

**The smaller sample size for assistant professors and postdocs has been added to the limitation section (LINE 503). We do speak to the lack of an intersectional lens in the limitations, though it is not necessarily due to sample size, but a function of our analytic plan (508 - 512).**

Supplemental Material, Participant Database: Please include the number of respondents per group in addition to the number of individuals queried.

**We added the number of participants per group in the supplemental tables S3-S9.**

Please include consistent line numbers throughout so reviewers don’t have to do math to make line comments.

**Sorry for that! The line number formatting is updated.**
