# Supplementary material for: Disproportionate impacts of COVID-19 on marginalized and minoritized early-career academic scientists
Source: PLoS One. 2022 Sep 13;17(9):e0274278. doi: 10.1371/journal.pone.0274278 (PMC9469975; doi:10.1371/journal.pone.0274278)
Supplement: Attachment — Submitted filename: Response to reviewers R3.docx [file pone.0274278.s003.docx]

**Response to Reviewers**

Reviewer #2: I appreciate the authors’ revisions, especially the inclusion of the models analyzing the graduate students and postdocs/assistant professors separately. I think those analyses enhance the insights from the study considerably. I have just a few minor additional comments and suggestions.

**Thank you for your thoughtful review and suggestions on this paper.**

Line 47: Please specify two or three of the “several” outcomes that were more negative for women, primary caregivers, underrepresented racial minorities, sexual minorities, and first-generation scholars. Otherwise this sentence is unnecessarily vague for an abstract.

**We included physical health symptoms and caretaking as examples in that section of the abstract.**

Line 121: While I appreciate the authors’ argument for the continued use of the word “prestige” in their response to reviews, it remains an inaccurate descriptor of these different institutions and unnecessarily implies author bias in importance and value of the different institutions surveyed in this study. I strongly recommend using the phrase “2011 National Research Council S-rankings”, since that is what the authors did, and is more transparent. “Prestige” is not a neutral term, and implies author bias to the reader that I think is unintended by the authors.

**We have changed the language on lines 121 and 160.**

Table 2: I am not sure “life problems” is the best term to use here. Perhaps life disruptions, or life challenges, the term the authors use in the discussion? Or something that doesn’t qualify physical health, mental health, or caretaking as “problems”?

**Thank you for this suggestion, we changed the title to “life challenges”**

Line 375: replace “the results above” with “Our results”. All of the study results indicate a wide array of consequences of the COVID-19 pandemic, not just the final paragraphs and tables of the results section.

**Thank you, we changed the language there.**

Line 415-417: It might be worth mentioning the anti-Asian sentiment in the US during 2021, as well as historic anti-Asian sentiment in the US (such as the Chinese Exclusion Act), when discussing the complex outcomes for Asian identifying scholars.

**Thank you for this suggestion. We’ve added that additional context to the discussion.**

For the figures: Since the columns for the three model sets (full, graduate student, and postdoc/assistant professor) are labeled, it is unnecessary and distracting to change the pattern and color. I suggest the authors keep the colors/pattern the same

**We opted to retain the pattern in the figures as they connect to the pattern in Tables 1 and 2. However, if the editor feels the pattern should be removed, we are happy to do so.**
